# The role of speech style, frequency, and density in recognition memory for spoken words

**DOI:** 10.3389/fpsyg.2024.1277624

**Published:** 2024-01-24

**Authors:** Anne Pycha, Tessa Culleton, Jae Yung Song

**Affiliations:** ^1^Department of Linguistics, University of Wisconsin-Milwaukee, Milwaukee, WI, United States; ^2^Department of English Language and Literature, Chung-Ang University, Seoul, Republic of Korea

**Keywords:** clear speech, word frequency, neighborhood density, recognition memory, models of memory

## Abstract

What determines whether listeners remember a spoken word? The Effortfulness Hypothesis claims that memory is modulated by a word’s intelligibility during real-time processing, while the Distinctiveness Hypothesis claims that it is modulated by a word’s distinguishing characteristics. We tested these differing predictions using American English words that varied along three dimensions known to affect both intelligibility and distinctiveness: speech style (clear versus casual), frequency (high versus low), and neighborhood density (high versus low). In a recognition memory experiment, participants (*n* = 66) listened to a set of study words, and then gave yes/no judgments to indicate whether or not they had heard the word earlier. Results showed that those words which exhibited distinctive characteristics – whether due to clear speech style, low frequency, or low density – were remembered better. The finding supports the Distinctiveness Hypothesis, suggesting that our capacity for remembering words relies on their distinctiveness, rather than on our capacity for recognizing them in real time.

## 1 Introduction

Our interactions with spoken language are affected by a wide variety of different sources. One factor is speaking style, in which talkers adjust their speech rate, pitch variation, and other acoustic parameters, in order to adapt to a particular situation (e.g., Smiljanić and Bradlow, 2009). Other factors include frequency of use, whereby certain phrases or words occur more often than others (e.g., Broadbent, 1967), and neighborhood density, which characterizes individual words according to how many similar-sounding words also exist in the lexicon (e.g., Luce, 1986).

Despite their diverse origins, there is strong evidence that all three of these factors affect listeners’ processing of speech stimuli in real time (for an overview, see Dossey et al., 2022). For example, numerous studies have shown that words are more intelligible when they are produced in a clear speech style, compared to a casual speech style (e.g., Picheny et al., 1985). Clear speech is a unique style that the speaker adopts in order to be better understood by a listener. Switching from conversational speech to clear speech gives rise to a number of acoustic changes, some of which are universal (Smiljanić and Bradlow, 2005) and some of which are specific to the speaker’s language (Cho et al., 2011). Universal modifications include slower speaking rates and more carefully articulated vowels, as indicated by an expanded vowel space (Picheny et al., 1986; Bradlow, 2002; Smiljanić and Bradlow, 2005). These types of vowel expansions are not typically present for speech produced in noise (Lu and Cooke, 2008; Davis and Kim, 2010; but see also Smiljanić and Gilbert, 2017), suggesting that clear speech is an intentional adaptation on the part of the speaker (Smiljanić, 2021).

Meanwhile, numerous studies have shown that listeners respond to words more quickly and accurately when they are frequent, compared to infrequent (e.g., Broadbent, 1967; Luce and Pisoni, 1998). Studies have also examined the role of phonological neighbors, which are words that differ from the target by the substitution of a phoneme (e.g., *pit, bat, bid* are neighbors of the word *bit*). These studies have shown that listeners respond to words more quickly and accurately when they are in low-density neighborhoods (i.e., with fewer neighbors), compared to high density neighborhoods (Luce and Pisoni, 1998; Vitevitch and Luce, 2016; Van Engen, 2017), presumably due to reduced competition from similar-sounding words.

Much more limited, however, is our understanding of how these factors affect listeners’ *memory* for words over the passage of time. While word frequency has been the topic of many studies on remembering, only a handful of studies have examined the role of neighborhood density and speech style. Among those few studies, Van Engen et al. (2012) reported higher rates of recognition memory for sentences produced with clear speech, compared to casual speech; this effect was present for semantically-normal as well as semantically-anomalous sentences. Keerstock and Smiljanić (2018) also reported higher rates of recognition memory for sentences produced with clear speech; this effect occurred for both L1 and L2 listening populations. In a follow-up study, Keerstock and Smiljanić (2019) conducted a cued-recall experiment, which is more difficult than a yes/no recognition memory task. Results showed that more words were recalled in the clear speech condition; as in Keerstock and Smiljanić (2018), this effect occurred for both L1 and L2 listening populations.

These findings about memory can be interpreted in at least two different frameworks. The first framework is the *Effortfulness Hypothesis* (Rabbitt, 1968, 1991; see also Van Engen and Peelle, 2014), which claims that when an item is easier to process in real time, more cognitive resources remain available for encoding. The logic is that since clear speech is more intelligible than casual speech, it requires less effort to process, and so listeners are able to devote more effort to memory storage. (The Perceptual Fluency hypothesis makes similar predictions, albeit without a direct appeal to encoding resources; Whittlesea et al., 1990; Goldinger et al., 1999).

However, the memory findings for clear speech could also be interpreted within a second framework called the *Distinctiveness Hypothesis* (Israel and Schacter, 1997; Schacter et al., 1997, 1998, 1999; Dodson and Schacter, 2001, 2002; see also Johnson and Raye, 1981). Within this framework, memory operates according to its own heuristic. The basic idea is that items with distinctive details are more likely to be remembered, compared to those without distinctive details, regardless of how such items are processed in real time. That is, certain items are distinctive enough that people respond using the heuristic, *If I had seen that, I would remember it* (Dodson and Schacter, 2001).

Applying this logic to the question at hand, utterances produced in clear speech are distinctive compared to those produced in casual speech. This is because clear speech is reserved for use only in certain types of circumstances, such as communicating with interlocutors who are hard-of-hearing. Meanwhile, for most everyday communication tasks, people use casual speech. Therefore, the Distinctiveness Hypothesis also predicts that people should remember clear speech utterances better than casual speech ones, but it does so for a different reason, namely that clear speech stands out as a singular type of event (e.g., *If I had heard an utterance pronounced in that deliberate way, I would have remembered it*).

For stimulus items that consist of full sentences produced in different styles (as in Keerstock and Smiljanić, 2018, 2019; Van Engen et al., 2012), it is difficult to distinguish between the Effortfulness versus the Distinctiveness Hypotheses, because they both make the same prediction, namely that there should be a memory advantage for clear speech. If we turn our attention to stimulus items that consist of individual words, however, there is one variable which may help to distinguish between the two frameworks, namely frequency. As noted above, people recognize frequent words more quickly and accurately than infrequent words in real time (e.g., Broadbent, 1967; Luce and Pisoni, 1998), which would suggest that they can devote more cognitive resources to encoding. Thus, the Effortfulness Hypothesis would predict that people should have better memory for frequent words. Yet this prediction is not confirmed: in recognition memory tasks, people actually remember frequent words more *poorly* than infrequent ones, as demonstrated by a number of studies over the years (Glanzer and Bowles, 1976; Glanzer and Adams, 1985, 1990; Joordens and Hockley, 2000).

The conflicting findings are puzzling and raise at least two possibilities. First, a “Modified” Effortfulness Hypothesis might argue that word-level variables, such as frequency and density, simply do not contribute to intelligibility (and by extension, to effortfulness) in the same way that speech style does. In most studies to date, word-level variables affect intelligibility *only* under special circumstances – for instance, when participants are under pressure to respond as quickly as possible, as in a typical lexical decision task. Indeed, Luce and Pisoni (1998) argued that if listeners were asked to classify stimuli in quiet with no time pressure, we would expect ceiling effects for all types of words. If frequent and infrequent words actually make similar processing demands under regular circumstances, then we do not expect them to differ in terms of effort. Under the Modified Effortfulness Hypothesis, then, there is no particular prediction for frequency effects (although presumably, the low-frequency advantage would need to be accounted for by appealing to some external factor), and therefore the previously-reported results for frequency do not pose a problem.

Alternatively, it is possible that effortfulness is not the primary factor at play in recognition memory, and that distinctiveness offers a better explanation. Just as clear utterances are distinctive compared to casual utterances, words that are infrequent are distinctive compared to words that are frequent, because they occur less commonly. Interpreted in this way, the Distinctiveness Hypothesis not only makes correct predictions for speech style, it also correctly predicts that people should remember infrequent words better than frequent ones (e.g., *If I had heard the rare word “puck,” I would have remembered it*) (Glanzer and Bowles, 1976; Glanzer and Adams, 1985, 1990; Joordens and Hockley, 2000).

One way to adjudicate between these two possibilities – an Effortfulness account on the one hand, versus a Distinctiveness account on the other – would be to examine whether the variables that make words easier to process also make them easier to remember. In doing so, it would be useful to examine speech style and frequency alongside an additional variable that also affects real-time processing of individual words. Neighborhood density is one such variable. The Effortfulness Hypothesis predicts that low-density words should be remembered better than high-density words, because there is an established processing advantage for low-density words (e.g., Vitevitch and Luce, 2016; Van Engen, 2017).

However, following the logic that we presented earlier, density may be similar to frequency in that it modulates intelligibility (and by extension, effortfulness) *only* under special circumstances (such as time pressure) and not in regular listening situations. If that is the case, then the Effortfulness Hypothesis would make no particular prediction for density effects, just as it would make none for frequency effects. By contrast, the Distinctiveness Hypothesis makes a clear prediction for density, regardless of the factors at play in real-time processing. Specifically, words that are low-density are distinctive compared to those that are high-density, because they contain sound combinations that occur less commonly in the lexicon. Therefore, the Distinctiveness Hypothesis predicts that people should remember low-density words better than high-density ones (e.g., *If I had heard the unusual-sounding word “pith,” I would have remembered it*).

Previous work offers mixed evidence with regard to these hypotheses. Several studies have shown that high-density words are remembered better than low-density ones (Guitard et al., 2023 and references cited therein), a finding which is not compatible with any of the hypotheses discussed above. However, these findings came from serial recall tasks, in which participants are asked to reproduce words in the same order in which they were initially presented. We are concerned with a different type of task, namely recognition memory, in which participants are presented with words individually and asked to indicate whether they are old or new. For recognition memory, at least one study has shown that low-density words are remembered better than high-density ones (Heathcote et al., 2006), a result that is consistent with the Distinctiveness Hypothesis.

The current study addresses these issues in a new recognition memory experiment using a stimulus set of isolated American English words that varied in speech style (clear versus casual), word frequency (high versus low), and neighborhood density (high versus low). Based on previous work, three different predictions are possible. The Effortfulness Hypothesis (Rabbitt, 1968, 1991; Van Engen and Peelle, 2014) would predict better recognition memory for words that are easier to process, namely words produced in clear speech style, high-frequency words, and low-density words. Second, if speech style affects intelligibility but word-level variables do not under certain circumstances, the Modified Effortfulness Hypothesis makes a narrower prediction, namely that we should expect better recognition memory only for clear speech. Third, the Distinctiveness Hypothesis predicts better recognition memory for words produced in a clear speech style, for low-frequency words, and for low-density words, regardless of the factors at play in real-time processing.

## 2 Materials and methods

### 2.1 Stimulus development

Target words were ninety-six CVC English words, evenly divided into four groups: high frequency/high density, high frequency/low density, low frequency/high density, and low frequency/low density. Frequency and density statistics were taken from the English Lexicon Project database (Balota et al., 2007). The mean log-transformed frequency (with base e) for high-frequency words was 10.42, and for low-frequency words was 6.63. The mean density for high-density words was 28, and for low-density words was 12. The words contained one of the six vowels /i, ɪ, æ, ɑ, ʌ, u/ and ended in voiceless codas (see Appendix 1 for a complete list of the stimuli).

Each word was recorded in both clear and casual styles, twice in each style. Words were recorded in a sentence context (“I will say X again”), and later excised. This was done because it was more natural to manipulate speech style when words were produced in a sentence context. The speaker was a female native speaker of the midwestern dialect of American English who had linguistic training, and who was already familiar with the concepts of clear versus casual speech styles.

### 2.2 Verification of stimuli

Before we proceed to the presentation of a recognition memory experiment, we will first present the results of two verification analyses. In the first analysis, we verified the effects of speech style, frequency, and density on the production of our word stimuli. Although frequency and density are lexical variables, they do not exist in a perceptual vacuum. They also affect speakers’ productions, creating phonetic differences in surface forms. To take one example, previous work has shown that the vowel spaces for high-frequency words tend to be more restricted, while vowel spaces for low-frequency words tend to be more expanded (Jurafsky et al., 2001; Munson and Solomon, 2004). Meanwhile, vowel spaces for high-density words tend to be more expanded, whereas those for low-density words tend to be more restricted (e.g., Munson and Solomon, 2004; Scarborough and Zellou, 2013 and references cited therein; Wright et al., 2004; but see also Gahl et al., 2012). Therefore, any experiment which uses naturally-spoken stimuli will not be able to strictly separate the effects of frequency and density from the effects of their phonetic manifestations, and the current experiment is no exception to this general issue.

In the second analysis, we verified the effects of speech style, frequency, and density on the intelligibility of our word stimuli. As discussed in the Introduction, clear speech, high frequency, and low density have been shown to make words easier to recognize. Thus, we wanted to know whether the same was also true for our own stimuli.

#### 2.2.1 Acoustic analysis of stimuli

To verify the effects of frequency, density, and speech style on phonetic forms, the recorded stimuli were acoustically analyzed by one of the authors in Praat (Boersma and Weenink, 2018), using the acoustic measures of vowel duration and vowel space area. Vowel onset and offset were defined as the beginning and end of the interval where F2 was clearly visible. Vowel formants (F1 and F2) were extracted from the midpoint of each vowel using a Praat script using the default parameters, and the accuracy of formant tracking was verified by visual inspection of formants. If the formant tracking in Praat did not reflect the actual formant bands seen in the spectrogram, various adjustments were made to improve the formant tracking, such as adjusting the number of formants counted by Praat. As there can be more than one way to form a polygon connecting the six vowel points (i.e., the F1 and F2 coordinates of six vowels), we used a convex hull to unambiguously define the vowel space area. The area of the convex hull was calculated using a built-in function of MATLAB (Mathworks, Inc.).

**Figure 1:**
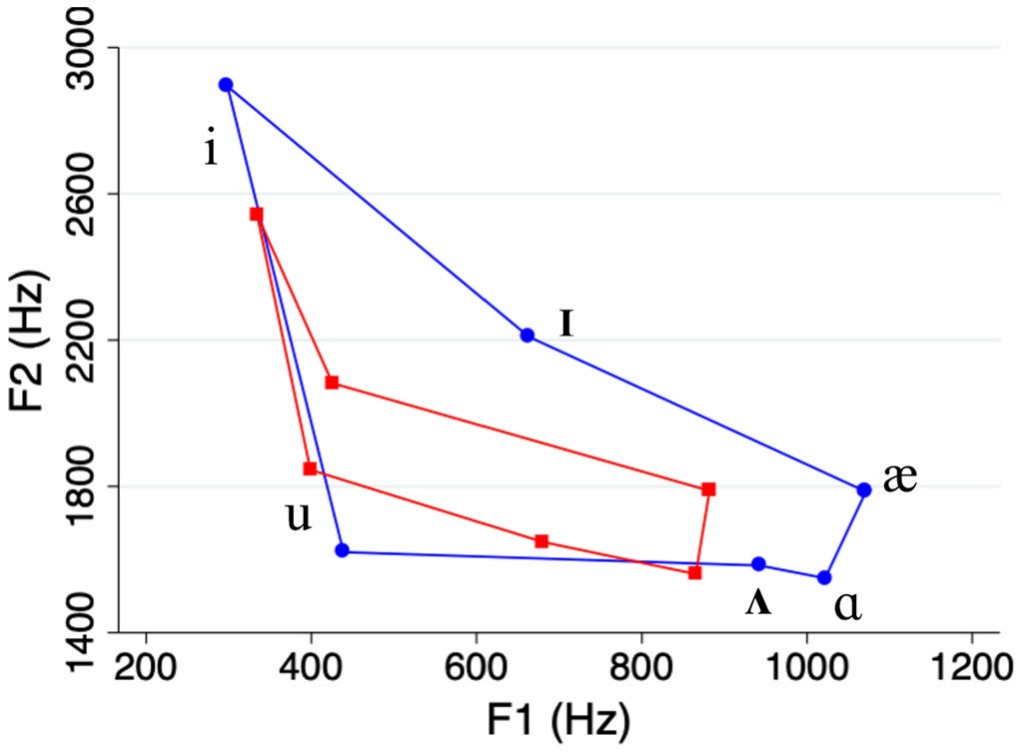
Vowel space in casual speech (red lines) compared to clear speech (blue lines).

**Figure 2:**
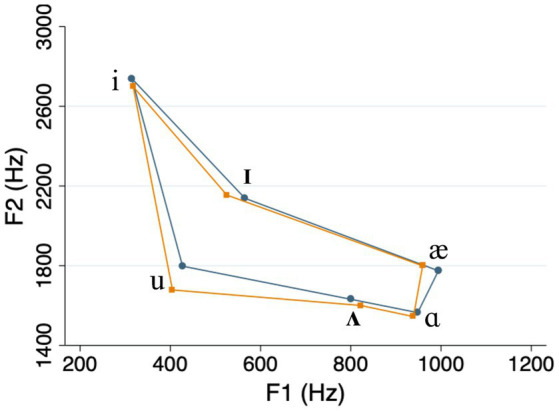
Vowel space in high frequency words (teal lines) compared to low frequency words (orange lines).

**Figure 3:**
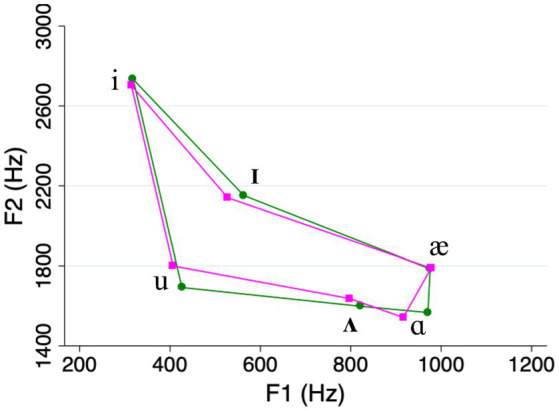
Vowel space in high density words (green lines) compared to low density words (magenta lines).

The acoustic analysis showed that our clear speech vowels were on average longer, and had expanded vowel space areas, compared to casual speech vowels (see Table 1 and Figure 1). This is consistent with the “universal” characteristics of clear speech that were mentioned in Section 1 (e.g., Smiljanić and Bradlow, 2005). Meanwhile, the differences in duration and vowel space area between high and low frequency words on one hand, and high and low density words on the other, were relatively small compared to what has been reported in previous studies (e.g., Munson and Solomon, 2004; see Table 1 and Figures 2, 3). We return to this point in the Discussion.

**Table 1:** Mean vowel duration (in msec) and vowel space area (in Hz^2^).

		Vowel duration (standard deviation)	Vowel space area
Speech style	Casual	92 (25)	227,887
	Clear	128 (32)	485,069
Frequency	High	110 (35)	325,277
	Low	110 (32)	357,374
Density	High	107 (28)	351,448
	Low	113 (39)	327,766

#### 2.2.2 Intelligibility of the stimuli

To verify the intelligibility of our recorded stimuli, we administered a brief task to a group of native speakers of American English (*n* = 26), none of whom participated in the main experiment. In an online Qualtrics survey of approximately 20 minutes, participants listened to each word, and were asked to type what they heard. The results showed that mean accuracy was greater for clear speech compared to casual speech (*β* = 1.03, std. error = 0.07, *z* = 15.13, *p* < 0.05), for frequent words compared to infrequent words (*β* = 0.56, std. error = 0.14, *z* = 3.92, *p* < 0.05), and for low-density words compared to high-density words (*β* = −0.53, std. error = 0.14, *z* = −3.68, *p* < 0.05). Thus, our stimuli conformed to the patterns of intelligibility that have been previously reported in the literature; namely, clear speech, high frequency, and low density facilitate real-time processing of spoken words. Note that in our verification study, these factors drive intelligibility differences even though the experimental set-up did not impose time pressure. This is likely due to the fact that the individual words were excised from full sentences, making the task of individual word transcription somewhat more difficult.

### 2.3 Procedure

The recognition memory experiment was implemented in a typical paradigm. In the study phase, participants heard a list of forty-eight stimuli, which was evenly balanced among clear versus casual styles, high versus low frequency words, and high versus low density words. The selection of the forty-eight study words from the pool of ninety-six words, and their presentation in either a clear or casual style, was balanced across participants using lists. The presentation of clear versus casual stimuli was blocked, and half of the participants heard clear speech first, while the other half heard casual speech first. Within each block, the order of stimuli was randomized for each participant. Participants were asked to try to remember the words.

In the test phase of the experiment, participants listened to a probe list of ninety-six stimuli. Half of the stimuli were old, meaning that the word had been presented during the study phase, while half of the stimuli were new. The participants’ task was to indicate “Yes” if they thought the word had occurred on the study list, otherwise “No.” Once a response was entered, the next trial began. Old stimuli were presented in the same style as at study, but with a non-identical token. For example, if the participant heard *cat_casual-token1_* during study, they would hear *cat_casual-token2_* during test. Stimuli were not blocked for speech style, and the presentation order was randomized for each participant. The experiment was conducted entirely online using Qualtrics software, and it took approximately 20 minutes to complete.

### 2.4 Participants

All participants (*n* = 66) were monolingual speakers of American English with no known speech or language impediments. No other exclusionary criteria were used. Participants were recruited through campus advertisements at University of Wisconsin-Milwaukee. Forty-four participants were female, seventeen were male, and five were non-binary; their mean age was 23.93 (8.02) years.

## 3 Results

Data from all participants was included in the analysis, and analyzed in aggregate form. Results were analyzed within a signal detection framework (Macmillan and Creelman, 2004), which involves using hit rates and false alarm rates to calculate the value of d-prime. D-prime indicates a participant’s overall sensitivity to old versus new words, and this sensitivity is composed of two parts. The first part is (correctly) remembering old items, and this is formalized as hit rate, or the proportion of old items that are recognized as old. The second part is (incorrectly) remembering new items, and this is formalized as false alarm rate, or the proportion of new items that are recognized as old. The value d-prime is calculated by subtracting the normalized probability of false alarms from the normalized probability of hits, and higher d-prime values indicate greater sensitivity to old versus new words.

Note that whenever a hit rate equals 1 or a miss rate equals 0, it becomes impossible to calculate the d-prime value correctly. When this occurred, we replaced rates of 1 with (n – 0.5)/n, and rates of 0 with 0.5/n, where *n* was the number of new or old trials (Macmillan and Kaplan, 1985). In preparing the data for analysis, d-prime values were calculated per participant, for each of the eight stimulus types (two speech styles × two frequency types × two density types). Descriptive results are shown in Table 2.

**Table 2:** Mean d-prime values (standard deviations) for recognition memory experiment.

		High frequency	Low frequency
Casual speech	High density	0.49 (0.77)	0.31 (0.75)
	Low density	0.75 (0.91)	0.77 (0.94)
Clear speech	High density	0.85 (0.77)	0.88 (0.68)
	Low density	1.06 (0.81)	1.37 (0.82)

Statistical results were analyzed using a linear mixed-effects model implemented with the lme function in the R package nlme. The outcome variable was d-prime. Predictor variables were speech style (casual vs. clear), word frequency (high vs. low), and neighborhood density (high vs. low), which were sum coded. The equation included a random intercept for participants. No random intercept for item was included, because the d-prime statistic is calculated over stimulus types, not individual items. Statistical results are shown in Table 3.

**Table 3:** Statistical analysis of d-prime values for recognition memory experiment.

	Val.	Std. Err	DF	*t*	*p*	
Style	0.23	0.03	455	7.50	0.00	*
Frequency	−0.02	0.03	455	−0.79	0.43	
Density	−0.18	0.03	455	−5.78	0.00	*
Style*Frequency	−0.06	0.03	455	−2.01	0.04	*
Style*Density	0.00	0.03	455	0.11	0.91	
Frequency*Density	0.06	0.03	455	1.98	0.04	*
Style*Frequency*Density	0.00	0.03	455	0.31	0.76	

**Figure 4:**
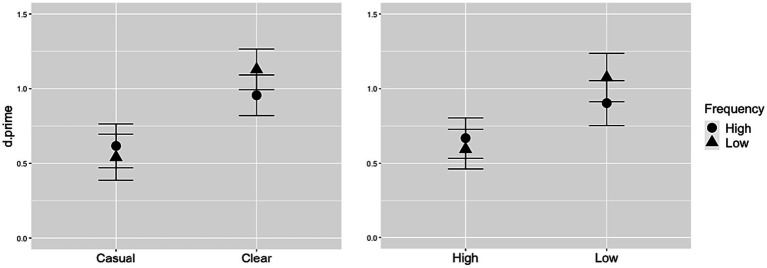
Interactions of frequency with speech style (left panel) and density (right panel). Whiskers depict standard error.

Statistical analysis showed an effect of speech style, whereby d-prime was significantly larger for clear speech than casual speech, and also an effect of neighborhood density, whereby d-prime was significantly larger for low-density words compared to high-density words. In addition, there were significant interactions between style and frequency, and between density and frequency, depicted in Figure 4. While low-frequency words generally had larger d-prime values than high-frequency words, this effect was greater in the clear speech condition, compared to casual (Figure 4, left panel) and also greater in the low-density condition, compared to high (Figure 4, right panel).

We conducted post-hoc power analyses using the pwr.f2.test() function from the pwr package in R, using values u (the numerator degrees of freedom) = 7 and v (the denominator degrees of freedom) = 3,160. For small, medium, and large effect sizes as defined by Cohen (2016), results yielded power values over 0.80, which indicates that the experiment was sufficiently powered.

## 4 Discussion

We examined the effects of speech style, frequency, and neighborhood density on recognition memory for spoken words. Our findings revealed that words produced in clear speech were remembered better than those produced in casual speech. Low-frequency words were remembered better than high-frequency words in certain conditions. Finally, low-density words were remembered better than high-density words. Broadly speaking, these results are most consistent with the Distinctiveness Hypothesis, which predicts better recognition memory for items that have distinctive traits, such as clear speech, low-frequency words, and low-density items. In the following paragraphs, we discuss each of our findings in turn, and consider their implications for different theories of recognition memory.

### 4.1 Speech style

The clear-speech advantage replicates previous studies (Van Engen et al., 2012; Keerstock and Smiljanić, 2018, 2019), and shows that the memory benefit for clear-speech stimuli can occur not just for full sentences, but also for individual words. On its own, this result does not adjudicate between different theories of memory, because it is compatible either with the Effortfulness Hypothesis, which predicts that clear speech is remembered better because it requires less effort at encoding, and the Distinctiveness Hypothesis, which predicts that clear speech is remembered better because it is distinctive compared to other types of speech. Nevertheless, this result is still notable insofar as certain elements of clear speech, such as an overall decrease in speaking rate, manifest themselves primarily in multi-word utterances. Our findings suggest that even when such elements are diminished (although not entirely absent, since our individual word stimuli were excised from full sentences), other acoustic characteristics, such as increases in vowel duration or changes to formant values, are sufficient to provide a memory benefit.

One previous study (Keerstock and Smiljanić, 2018) had suggested that the benefits of clear speech were activated through relatively deep, rather than shallow, linguistic processes. In the study phase of this experiment, sentences were produced auditorily, in either clear or conversational speech. In the test phase, however, sentences were presented orthographically. Results showed that participants remembered sentences presented in clear speech better than those presented in conversational speech, and the fact that the benefit persisted across modalities was interpreted as indicative of deep linguistic processing. In the current study, both study and test items were always presented auditorily; crucially, however, the individual tokens were not identical across the two presentations. This means that, when recognizing a previously-heard words as old, participants could not rely on episodic recognition of surface features, but rather had to draw upon a word-level encoding of the stimulus. Thus, the current study is broadly consistent with Keerstock and Smiljanić (2018) in showing that memory traces for clear speech appear to be activated at a level which is abstracted from the input stimulus.

### 4.2 Frequency

Although there was no main effect of word frequency on recognition memory, frequency did exhibit significant interactions with other factors. Specifically, low-frequency words increased recognition memory for words that were already comparatively easy to remember, namely those which were produced in clear speech or had low neighborhood densities. Thus, to the extent that we see frequency effects in the current study, they are consistent with previous studies of recognition memory, which report an advantage for low-frequency words (Glanzer and Bowles, 1976; Glanzer and Adams, 1985, 1990; Joordens and Hockley, 2000).

Crucially, the frequency findings are compatible with the Distinctiveness Hypothesis, which predicts better recognition memory for low-frequency words, compared to high-frequency words. By contrast, they are not compatible with the Effortfulness Hypothesis, which would predict the opposite pattern.

In the Introduction, we had considered a scenario, the Modified Effortfulness Hypothesis, in which word-level factors do not contribute to effort. The logic was that, under regular listening circumstances with no time pressure, we do not expect intelligibility differences for low versus high frequency words, and therefore we do not expect effortfulness differences, either. Within such a scenario, the Modified Effortfulness Hypothesis would essentially make no prediction for frequency effects. However, our results do not provide support for this logic. Recall that the intelligibility analysis, reported in Section 2.2.2, showed that our stimuli did indeed exhibit significant differences in accuracy: for example, the overall accuracy rate for high-frequency words was significantly greater than low-frequency words. This suggests that, at least for the stimuli used here, the processing advantage for high-frequency words did extend beyond situations of time pressure, and that the Modified Effortfulness Hypothesis is not tenable.

### 4.3 Density

Our results showed a significant effect of density, whereby low-density words exhibited better recognition memory than high-density words. This finding is consistent with previous work that manipulated neighborhood density in a recognition task (Heathcote et al., 2006). Taken in isolation, the results for density would be compatible with either the Effortfulness Hypothesis or the Distinctiveness Hypothesis, both of which predict a recognition memory advantage for low-density words. Taken together with the findings on frequency, however, it becomes difficult to support either version of the Effortfulness Hypothesis. Recall that the Effortfulness predicts an advantage for high-frequency words in recognition memory; as discussed in Section 3, this prediction was not met. A Modified Effortfulness Hypothesis considered the possibility that density has no effect on intelligibility (and by extension, effortfulness), but the results from our intelligibility task do not support for this idea, because accuracy was significantly higher for low-density words than for high-density words. Thus, as was the case for high-frequency words, the processing advantage for the low-density words in our study did extend beyond situations of time pressure, and therefore the Modified Effortfulness Hypothesis is not tenable.

### 4.4 Remaining questions and future directions

#### 4.4.1 Measurement of distinctiveness

The current study represents a first step toward exploring the role of distinctiveness in recognition memory for spoken words. In doing so, we have employed very basic working definitions of what it means to be “distinct,” reasoning that clear speech is distinct because most conversations occur in casual speech, that low-frequency words are distinct because they occur less commonly than high-frequency words, and that low-density words are distinct because their phonological neighborhoods are less crowded than those of high-density words. For the future, a next step would be to measure distinctiveness in a more direct manner – for example, by asking listeners to rate the distinctiveness of individual words on a Likert scale – and to correlate these ratings with recognition memory results. Such results would indicate whether listeners’ actual *experience* of distinctiveness leads to better remembering, and provide an important corroboration for the Distinctiveness Hypothesis.

#### 4.4.2 Individual differences

The act of remembering varies a great deal from one individual to the next (Bors and MacLeod, 1996). For recognition memory, one of the most relevant factors is age: older adults typically exhibit lower d-prime values than younger adults, as well as an increased tendency to label items as “new” (Fraundorf et al., 2019). Even among people of similar ages, however, individuals may still require differing amounts of evidence before committing to an “old” decision (Kantner and Lindsay, 2012) – and presumably, some of this evidence comes from an item’s distinctiveness. In the current study, participants were younger adults (mean age 23.93 [8.02] years) who may have nevertheless exhibited individual differences that we have not examined here. Future work could investigate this issue, for example, by testing for links between individuals’ overall memory capacity, on the one hand, and recognition rates for distinctive words, on the other.

#### 4.4.3 Phonetic realizations

In Section 2.2, we noted that speech style, frequency, and density can affect the phonetic realizations of spoken words (Jurafsky et al., 2001; Munson and Solomon, 2004; Wright et al., 2004; Gahl et al., 2012; Scarborough and Zellou, 2013), making it potentially difficult to isolate the effects of these variables. This would pose a conundrum for any speech researcher, yet our current results do offer some clarity in this regard. To begin with, in our own stimuli, the vowel-space differences between different word types were relatively small (see Figures 2, 3), suggesting a diminished role for phonetic differences. Even to the extent that such differences did exist, however, they cannot fully account for our pattern of results. For example, as we pointed out earlier, vowel spaces for high-density words tend to be more expanded than those for low-density words. If hyper-articulated vowels are more distinctive for listeners in recognition memory, this would predict that listeners should remember high-density words better. But this is clearly not the case. Instead, our results show that listeners remember low-density words better, suggesting that their structural distinctiveness exerts an influence independently of lower-level phonetic effects. Of course, the only way to completely separate lexical versus phonetic effects would be to conduct experiments using printed or synthesized-speech stimuli. Although both techniques introduce their own additional confounds, such approaches could be explored in future work.

#### 4.4.4 Elaborating the distinctiveness hypothesis

There are several potential avenues for further developing the Distinctiveness Hypothesis as it relates to memory for spoken words. As we have discussed, our overall results showed better recognition memory for words produced in clear speech, for low-frequency words in certain conditions, and for low-density words. Importantly, there is more than one mechanism by which these distinctiveness advantages could conceivably originate. One potential mechanism is better recognition of words that were actually heard (*“shuck” is an unusual-sounding word, so I definitely remember hearing it earlier*), which would produce higher hit rates. Within a signal detection framework in which the listener’s task is to detect a signal amidst noise, this would mean that distinctive words contribute to a stronger signal. Another potential mechanism is reduced recognition of words that were not heard (*“gaffe” is an unusual-sounding word, so I am certain that I did not hear it earlier*), which would produce lower false alarms. Within a signal detection framework, this would mean that distinctive words contribute reduced noise.

For the moment, we speculate that both factors may be at play. Recall that there was an effect of speech style in our analysis of the variable d-prime. If we break this variable into its component parts, we see that hit rates were higher for clear (0.67 [0.20]) versus casual (0.58 [0.25]) stimuli and also that false alarm rates were lower for clear (0.33 [0.20]) versus casual (0.38 [0.21]) stimuli. To take another example, there was also an effect of density in our analysis of d-prime. Breaking this down, we see a similar pattern, whereby hit rates were higher for low-density (0.66 [0.23]) versus high-density (0.59 [0.23]) words and false alarm rates were also lower for low-density (0.33 [0.20]) versus high-density words (0.38 [0.20]). (Recall that for frequency, there was an effect for d-prime only when it interacted with other factors, so we did not break down those results further). Future research should help to illuminate the exact conditions under which people benefit from a stronger signal versus reduced noise, and thereby more finely characterize the role that distinctiveness plays in memory for spoken words.

While it is relatively straightforward to apply a “distinctiveness” criterion to individual words, it is less clear how to apply it to entire sentences. Indeed, previous findings showing that listeners remember semantically normal sentences better than semantically anomalous sentences (Van Engen et al., 2012), and that L1 listeners remember sentences better than L2 listeners (Keerstock and Smiljanić, 2018), would be difficult to account for in this framework. However, it is possible that different factors influence memory for individual words on the one hand, versus multi-word sentences on the other. Related to the L1 versus L2 sentence findings, for example, Francis and Gutiérrez (2012) showed that Spanish-English bilinguals exhibit better recognition memory for words in their non-dominant language, compared to their dominant language, a result that is consistent with the Distinctiveness Hypothesis. To sort through these issues in future studies, a potential starting point could be the work of Garnham (1981), who argued that we represent sentences using mental models of events, rather than linguistic expressions *per se*. It stands to reason that the same may not be true for individual words.

Memory is a complex human behavior. Indeed, as Schacter (1996, p. 6) notes, “memories are records of how we have experienced events, not replicas of the events themselves.” As a consequence of this complexity, patterns of remembering can differ based upon the task at hand. In previous work and in the current study, for example, the advantage for low-frequency words was found for recognition memory tasks. These tasks gauge an implicit feeling of familiarity, because participants give a simple old/new response to words that are presented individually. However, the low-frequency advantage has *not* been found for recall tasks (e.g., Balota and Neely, 1980; for review, see Popov and Reder, 2020). These tasks require explicit recollection, because participants must remember the previously-presented words without hearing or seeing them again at test. A similar task asymmetry occurs for density: our recognition results show an advantage for low-density words, but recall tasks do not exhibit this advantage (e.g., Guitard et al., 2023).

Meanwhile, this task asymmetry is not apparent for speech style, where previous work has shown that the clear-speech advantage occurs in both types of tasks, namely in recognition memory as well as in cued recall (Keerstock and Smiljanić, 2019). This suggests that the nature of the memory benefit provided by clear speech may be distinct from that of low frequency and low density, in spite of the fact that all three characteristics are distinctive. In future studies, we hope to broaden our inquiry into the nature of human memory, and to compare how speech styles, frequency, and density affect different types of remembering tasks. We also aim to explore how our findings might generalize to real-world situations. For example, the purposeful use of distinctive words (rather than less distinctive ones) could potentially be useful when teaching or giving instructions.

While we have presented evidence in favor of the Distinctiveness Hypothesis, the possibility remains that, at least for certain cases, both effort and distinctiveness may be at play simultaneously. In the current study, for example, clear speech and low-density words showed significant effects on recognition memory, whereas frequency effects were found only in certain conditions. Interestingly, while the Effortfulness and Distinctiveness Hypotheses make different predictions for frequency, they make similar predictions for clear speech and low-density words. This suggests the possibility that words which are both less effortful *and* more distinctive exhibit additive effects on recognition memory, which can be explored in future work.

## 5 Conclusion

Memory is a complex cognitive undertaking, even when we consider the relatively simple task of remembering a single spoken word. In the current study, we examined speech style, a factor that is typically operative at the utterance level, as well as frequency and neighborhood density, which are operative at the level of individual words. Our results showed that those words which exhibited distinctive characteristics – whether due to clear speech style, low frequency, or low density – were remembered better. This finding is readily accounted for by the Distinctiveness Hypothesis, and suggests that our human capacity for remembering words which were spoken in the past need not crucially rely on our capacity for recognizing them in real time. Rather, memory may operate according to its own independent heuristic. *If I had heard that rare, unusual-sounding word pronounced in that deliberate way, I would have remembered it!*

## Data availability statement

The raw data supporting the conclusions of this article will be made available by the authors, without undue reservation.

## Ethics statement

The studies involving humans were approved by the Institutional Review Board (IRB) at the University of Wisconsin-Milwaukee. The studies were conducted in accordance with the local legislation and institutional requirements. The participants provided their written informed consent to participate in this study.

## Author contributions

AP: Conceptualization, Data curation, Formal analysis, Funding acquisition, Investigation, Methodology, Writing – original draft, Writing – review & editing. TC: Conceptualization, Data curation, Investigation, Methodology, Writing – original draft, Writing – review & editing. JS: Conceptualization, Data curation, Formal analysis, Funding acquisition, Investigation, Methodology, Writing – original draft, Writing – review & editing.

## APPENDIX 1

Stimulus words.

**Table:**

High frequency High density	High frequency Low density	Low frequency High density	Low frequency Low density
back	chief	beak	botch
beat	cup	coop	cuff
bit	dish	cot	dash
boot	duke	dip	ditch
buck	Dutch	gut	fuss
bus	fish	hick	gaffe
cat	half	hoot	geese
cut	josh	hut	goof
duck	juice	kip	goose
fit	kiss	knit	goth
got	path	pap	gush
hit	shop	peat	hiss
hot	such	pip	hush
kit	teach	pock	miff
pack	teeth	puck	niche
peak	that	pup	pith
pick	thick	putt	posh
pop	this	sap	puff
seek	thus	seep	sheaf
shut	touch	sip	sheath
sit	tough	tack	shuck
soup	watch	toot	thatch
suck	youth	tot	tiff
suit	zip	tuck	tooth
